# Canine tick-borne pathogens in Cyprus and a unique canine case of multiple co-infections

**DOI:** 10.1016/j.ttbdis.2016.12.006

**Published:** 2017-03

**Authors:** Charalampos Attipa, Chelsea A.E. Hicks, Emily N. Barker, Vasiliki Christodoulou, Kyriaki Neofytou, Mathios E. Mylonakis, Victoria I. Siarkou, Elpida I. Vingopoulou, Francesca Soutter, Dimosthenis Chochlakis, Anna Psaroulaki, Kostas Papasouliotis, Séverine Tasker

**Affiliations:** a Diagnostic Laboratories, Langford Vets, School of Veterinary Sciences, University of Bristol, Langford, UK; b Cyvets Veterinary Center, Paphos, Cyprus; c Veterinary Services of Cyprus, Nicosia, Cyprus; d Companion Animal Clinic, School of Veterinary Medicine, Faculty of Health Sciences, Aristotle University of Thessaloniki, Thessaloniki, Greece; e Laboratory of Microbiology and Infectious Diseases, School of Veterinary Medicine, Faculty of Health Sciences, Aristotle University of Thessaloniki, Thessaloniki, Greece; f Department of Pathology and Pathogen Biology, Royal Veterinary College, Hertfordshire, UK; g Laboratory of Clinical Bacteriology, Parasitology, Zoonoses and Geographical Medicine, Medical School, University of Crete, Greece

**Keywords:** CTBP, canine tick-borne pathogens, qPCR, quantitative polymerase chain reaction, VBD, vector-borne disease, *Ehrlichia canis*, *Anaplasma platys*, *Hepatozoon canis*, *Babesia vogeli*, *Mycoplasma haemocanis*, Canine tick-borne pathogens, Cyprus

## Abstract

Canine tick-borne pathogens such as *Ehrlichia canis* and *Hepatozoon canis* are widespread in the Mediterranean basin but have never been reported or investigated in Cyprus. We describe herein the presence of canine tick-borne pathogens in three dogs with clinical signs compatible with vector-borne diseases from Paphos area of Cyprus. Molecular and phylogenetic analysis revealed the presence of *E. canis*, *Anaplasma platys*, *H. canis*, *Babesia vogeli* and *Mycoplasma haemocanis* in Cyprus. One dog co-infected with *E. canis*, *H. canis*, *B. vogeli* and *M. haemocanis* is, to the best of our knowledge, the first report of this multiple co-infection in dogs. The tick-borne pathogens reported in the current study should be considered in the differential diagnoses in dogs exposed to ticks in Cyprus.

## Graphical abstract

**Figure:**
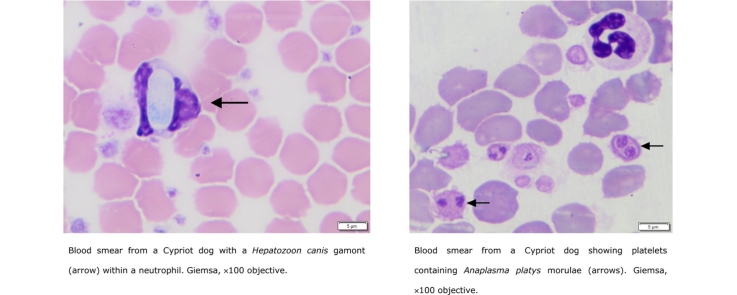

## 1 Introduction

Cyprus is a European Union member island state situated in the eastern Mediterranean basin (35°10′N and 33°22′E) with a temperate climate. Several tick-borne pathogens are endemic in domestic ruminants (goats, sheep and cattle) (Chochlakis et al., 2012, Chochlakis et al., 2009, Psaroulaki et al., 2009), wild animals (mouflon, fox and hare) (Chochlakis et al., 2012, Ioannou et al., 2011, Psaroulaki et al., 2014) and migratory bird populations of the island (Ioannou et al., 2009). *Rhipicephalus sanguineus* sensu lato, the main vector for several canine tick-borne pathogens (CTBP), is the most common tick species found in dogs in Cyprus (Chochlakis et al., 2012, Dantas-Torres, 2010, Le Riche et al., 1974). The only vector-borne disease (VBD) previously reported in dogs in Cyprus is leishmaniosis (Mazeris et al., 2010). Up to now, no studies have yet reported the presence of CTBP in Cypriot dogs.

This report describes, for the first time, the molecular detection of *Ehrlichia canis*, *Anaplasma platys*, *Hepatozoon canis*, *Babesia vogeli* and *Mycoplasma haemocanis* in three naturally-infected dogs from Cyprus with clinical signs compatible with VBD.

## 2 Material and methods

Three dogs from Cyprus, designated as Cases 1, 2 and 3, with no overseas travel history and with clinical signs compatible with VBD, were admitted to Cyvets Veterinary Center, at Paphos, in 2013 and included in this study.

At clinical presentation, as part of diagnostic procedures, blood samples were collected into EDTA and heparin tubes and analysed by an automated haematology impedance analyzer (Vet ABC, Scil, Viernheim, Germany) and by a VetScan VS2 chemistry analyzer (Abaxis, USA), respectively. Blood smears prepared on glass slides at the time of blood collection were air-dried and stained with Giemsa (Merck, Darmstadt, Germany) and examined by light microscopy. Additionally, plasma samples were evaluated for the presence of antibodies against *E. canis*/*E. ewingii*, *Borrelia burgdorferi* sensu lato, *A. platys/Anaplasma phagocytophilum* and the presence of *Dirofilaria immitis* antigen using the SNAP 4Dx^®^ Plus test (IDEXX Laboratories, Westbrook, Maine, USA). Urine samples were collected via cystocentesis and analysed using the Multistix 10SG urine reagent strips (Siemens AG, Munich, Germany).

Following diagnostic testing and with written owner consent, surplus stored EDTA-blood was used retrospectively for this study. DNA was extracted from 100 mL of EDTA-blood using a commercial kit (NucleoSpin^®^ Blood, Machery-Nagel, Germany) according to the manufacturer's instructions. Real-time quantitative polymerase chain reaction (qPCR) assays were used to investigate for the presence of haemotropic mycoplasmas (*M. haemocanis* and ‘*Candidatus* Mycoplasma haematoparvum’), *B. burgdorferi* sensu lato and *Bartonella henselae,* conventional PCRs were used to evaluate for *Ehrlichia/Anaplasma* spp., *Hepatozoon* spp., *Babesia* spp. and *Leishmania* spp. infection, and a nested *E. canis*-specific PCR assay was also performed (Table 1) (Barker et al., 2010, Christodoulou et al., 2012, Dawson et al., 1996, Demaerschalck et al., 1995, Inokuma et al., 2002, Matjila et al., 2008, Oosthuizen et al., 2008, Parola et al., 2000, Robinson et al., 2010). To confirm the presence of amplifiable DNA and absence of PCR inhibitors, all qPCR reactions were duplexed with an internal control for glyceraldehyde-3-phosphate dehydrogenase. For each PCR run, positive controls using DNA from known infected dogs and negative controls using water were included.

After conventional PCR amplification, the amplicons from any positive *Ehrlichia/Anaplasma* spp., *Hepatozoon* spp., *Babesia* spp. and *E. canis* PCR assays were purified (ExoSAP-IT, Affymetrix, USB, Cleveland, Ohio, USA) and the DNA sequenced on both strands using the respective forward and reverse PCR primers. For each DNA amplicon forward and reverse sequences were assembled, and a consensus sequence constructed, using ClustalW in Bioedit. DNA sequences were deposited in GenBank and checked for identity against previously deposited sequences, using NCBI BLAST (Altschul et al., 1990). Sequences obtained in this study were aligned to selected sequences of the respective genes, from the same and related species of organism, previously deposited in GenBank using ClustalW. Phylogenetic trees were constructed using the maximum likelihood program, corrected for nucleotide substitutions by the Kimura-2 parameter model, in MEGA version 6 (Tamura et al., 2013). The data set was resampled 1000 times to generate bootstrap percentages.

## 3 Case reports

### 3.1 Case 1

A 1.5-year-old, entire male, mixed-breed dog presented with a 6-day history of depression and anorexia. The dog lived outdoors in rural Paphos-area. Physical examination revealed pale mucous membranes with widespread petechial haemorrhages. Body condition score was 4/9. Haematology showed a mild normochromic normocytic anaemia (haematocrit [Hct] 34.5%; reference interval [RI] 44–57%), monocytosis (1.4 × 10^9^/L; RI 0.0–0.5 × 10^9^/L), lymphocytosis (4.0 × 10^9^/L; RI 1.0–3.6 × 10^9^/L) and a moderate thrombocytopenia (65 × 10^9^/L; RI 200–460 × 10^9^/L), while no abnormalities were detected on biochemistry and urine analysis. Giemsa stained peripheral blood smear examination revealed the presence of gamonts of *Hepatozoon* spp. intracellularly in 0.2% of neutrophils. Seropositivity for *E. canis/E. ewingii* was also detected. Based on the physical examination, haematological abnormalities and serological results, a diagnosis of *Hepatozoon* spp. infection was established, with presumptive co-infection with *Ehrlichia* spp. Treatment with doxycycline (Ronaxan, Merial, Lyon, France; 5 mg/kg orally twice daily for 28 days) was given. Abnormalities were not detected on repeat physical examinations, haematological analysis and blood smear examination on days 28 and 134 following treatment initiation. Subsequent PCR on pre-treatment stored blood samples and sequencing analysis confirmed both *E. canis* and *H. canis* infection and revealed concurrent infection with *B. vogeli* and *M. haemocanis*.

### 3.2 Case 2

A 7-year-old, entire female, mixed-breed dog presented with a 4-month history of lethargy, weakness, anorexia and weight loss. The dog lived outdoors in rural Paphos-area. Physical examination showed peripheral lymphadenomegaly, a body condition score of 2/9, pale mucous membranes, epistaxis and bilateral blepharitis, conjunctivitis with mucopurulent ocular discharge and anterior uveitis. Haematology revealed a moderate normochromic and normocytic anaemia (Hct 19.0%; RI 44–57%), leucopenia (4.2 × 10^9^/L; RI 6.0–12.0 × 10^9^/L), lymphopenia (0.6 × 10^9^/L; RI 1.0–3.6 × 10^9^/L) and thrombocytopenia (118 × 10^9^/L; RI 200–460 × 10^9^/L). Biochemical abnormalities included hyperproteinaemia (94.5 g/L; RI 54–82 g/L), hyperglobulinaemia (74 g/L; RI 23–52 g/L) and hypoalbuminaemia (20 g/ L; RI 25–44 g/L). No abnormalities were detected on urine analysis. Blood smear examination revealed 0.8% of neutrophils to be parasitized by gamonts of *Hepatozoon* spp. Serology was positive for *E. canis/E. ewingii*. *Leishmania* amastigotes were noted in macrophages and extracellularly on fine needle aspiration cytology (Giemsa stained) of the right submandibular lymph node. A diagnosis of clinical leishmaniosis with concurrent *Hepatozoon* spp. infection and presumptive co-infection with *Ehrlichia* spp. was made. Further investigation and treatment for leishmaniosis was discussed, however, the owners elected euthanasia and declined necropsy. Subsequent PCR on pre-treatment stored blood samples confirmed *Leishmania* spp. infection while PCR and sequencing analysis revealed the presence of *E*. *canis* and *H*. *canis*.

### 3.3 Case 3

A 1-year-old, neutered female, mixed-breed dog presented with a 2-day history of decreased appetite and right hindlimb lameness. The dog lived partially outdoors in urban Paphos. Clinical examination revealed right stifle soft tissue swelling and pyrexia (39.3 °C). Haematology showed a moderate normochromic and normocytic anaemia (Hct 18.9%; RI 44–57%) and thrombocytopenia (64 × 10^9^/L; RI 200–460 × 10^9^/L). Blood smear examination revealed *A. platys* morulae within most of the platelets. No abnormalities were detected on biochemistry and urine analysis. Cytological examination of the synovial fluid (Giemsa stained) from the right stifle identified a neutrophilic arthritis. No other joints were sampled. Serology, using the SNAP 4Dx^®^ test, was unremarkable. An initial diagnosis of thrombocytotropic anaplasmosis was made and the dog was treated with doxycycline (Ronaxan, Merial, Lyon, France; 5 mg/kg orally twice daily for 28 days). On days 28 and 134 following treatment, the dog was described as being healthy by the owner via telephone communication, but repeat examination was declined. Subsequent PCR on stored pre-treatment blood samples and sequencing analysis confirmed *A. platys* infection.

## 4 Results

### 4.1 Sequence and phylogenetic analysis

The sequencing results of products derived from positive *Ehrlichia/Anaplasma* spp., *Hepatozoon* spp., *Babesia* spp. and *E. canis* PCR assays, together with the lengths of the sequence data obtained, are shown in Table 1, alongside the GenBank accession numbers for the generated sequences.

**Fig. 1:**
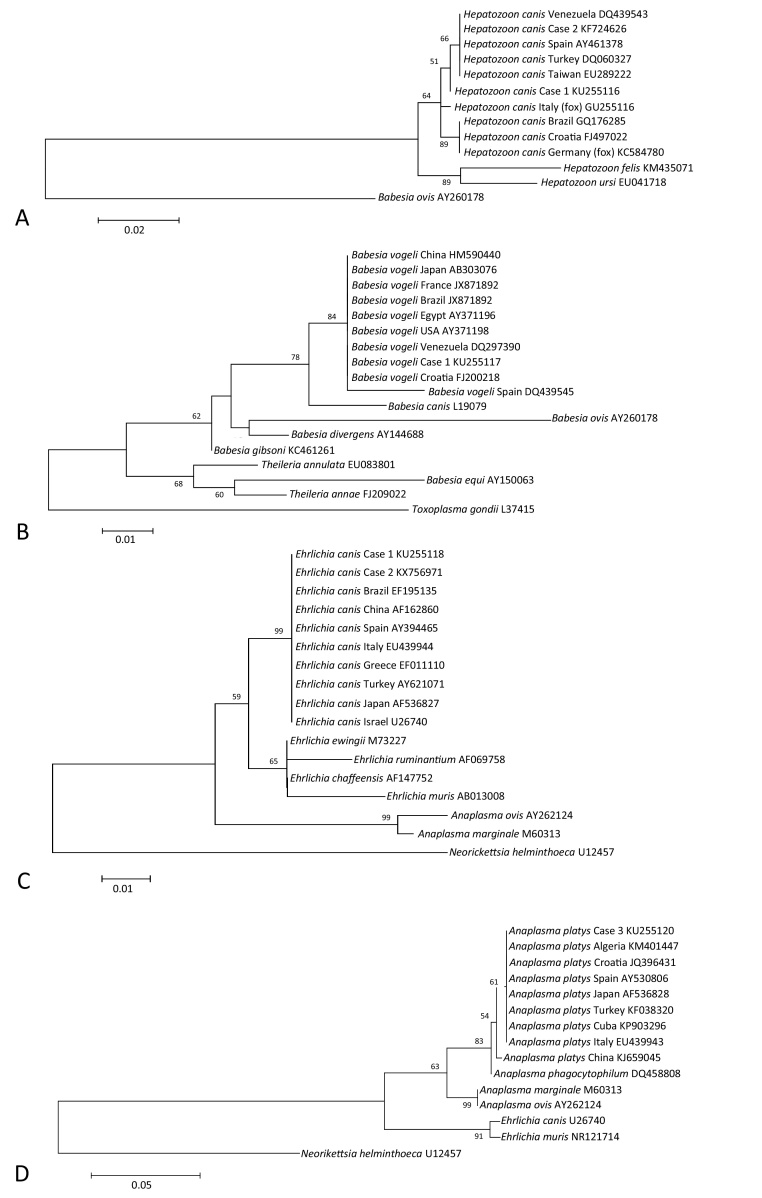
Phylogenetic analyses of *Hepatozoon canis* (A), *Babesia vogeli* (B), *Ehrlichia canis* (C), and *Anaplasma platys* (D) gene fragments amplified from cases in this study (as described in Table 1) and selected gene fragments available from GenBank, and those of related organisms. Phylogenetic trees were constructed by the maximum likelihood method. The data sets were resampled 1000 times to generate bootstrap percentage values, and values greater than 50% are given at the nodes of the tree.

On phylogenetic analysis, all of the *H*. *canis* sequences compared clustered together, including those from Cases 1 and 2, in a single clade, separate from *H. felis* and *H. ursi* (Fig. 1A). All *B. vogeli* sequences compared, including that from Case 1, also clustered together in a single clade, distinct to other *Babesia* and *Theileria* spp. (Fig. 1B). All *E. canis* sequences compared, including those from Cases 1 and 2, clustered together in a single clade supported, distinct to other *Ehrlichia* and *Anaplasma* spp. (Fig. 1C). Most of the *A. platys* sequences compared, including that from Case 3, clustered closely together in a single clade (Fig. 1D), although one *A. platys* sequence from China branched separately, albeit with a relatively low bootstrap value.

## 5 Discussion

Canine tick-borne pathogens such as *E. canis*, *H. canis*, *B. vogeli* and *M. haemocanis* have been documented in several European countries especially in the Mediterranean region where the temperate climate sustains the presence and activity of *R. sanguineus* sensu lato which is considered the major vector for the transmission of these pathogens (Baneth, 2011, Novacco et al., 2010, Sainz et al., 2015, Solano-Gallego and Baneth, 2011). Two previous studies have described the canine tick flora found on dogs in Cyprus, with *R. sanguineus* sensu lato comprising 87.5-89.4% of ticks collected (Chochlakis et al., 2012, Le Riche et al., 1974). No previous studies have investigated CTBP in Cypriot dogs.

Case 1 was diagnosed with likely acute monocytic ehrlichiosis, and even though it was co-infected with three additional CTBP (*H. canis*, *B. vogeli* and *M. haemocanis*) the clinical and haematological outcome did not appear to be worsened. Co-infection has previously been demonstrated in Greek dogs; one study demonstrated that 65% of dogs in Greece, with naturally-occurring monocytic ehrlichiosis, were also seropositive to *H. canis* (Mylonakis et al., 2005). In the same study, the prevalence of *H. canis* parasitaemia and the number of circulating gamonts were low, and *H. canis* infection did not appear to exacerbate the clinical manifestations or the haematological abnormalities of monocytic ehrlichiosis. In Case 1, there was a low degree of *H. canis* parasitaemia (<5%), which might suggest limited involvement in the clinical signs of the dog (Baneth, 2011). The fact that there was no cytological evidence of *H. canis* parasitaemia after follow-up cytological review of the peripheral blood smear, coupled with the complete clinical and haematological recovery following doxycycline treatment, suggests that this infection was not a critical determinant in the clinical and clinicopathologic outcome. The clinical relevance of the *B. vogeli* and *M. haemocanis* infections in Case 1 is uncertain, as both pathogens tend to affect splenectomized dogs more severely, and their clinical and haematological abnormalities may overlap with those of monocytic ehrlichiosis (Chalker, 2005, Solano-Gallego and Baneth, 2011). Additionally, doxycycline treatment should be effective against *M. haemocanis* (Chalker, 2005). To the authors’ knowledge this is the first report of a dog being co-infected with *E. canis*, *H. canis*, *B. vogeli* and *M. haemocanis*.

Dual *E. canis* and *Leishmania* spp. infections, as seen in Case 2, are commonly reported and tend to have prominent clinical signs and slower recoveries compared to dogs with single infections of either pathogen (Di Loria et al., 2009, Mekuzas et al., 2009). Since these diseases overlap substantially in their pathology (Mylonakis et al., 2008), the clinical and clinicopathologic abnormalities seen in this case could have been attributed to either *E. canis* or *Leishmania* spp. infection. In a previous study, dogs that presented with epistaxis, as in Case 2, and with mucosal pallor and pancytopenia, were more likely to be affected by monocytic ehrlichiosis, as opposed to the presence of peripheral lymphadenomegaly, which was more indicative of leishmaniosis (Mylonakis et al., 2008). Similar to Case 1, the *H. canis* infection in Case 2 was likely to be an incidental finding and most likely of limited clinical significance.

Case 3 was diagnosed with thrombocytotropic anaplasmosis, with thrombocytopenia being the typical clinicopathologic finding of *A. platys* infection. Although a definitive mode of transmission has yet to be demonstrated for *A*. *platys*, *R*. *sanguineus* sensu lato is thought to be the most likely vector (Sainz et al., 2015). Unlike the well-recognised *A. phagocytophilum*-induced immune-mediated polyarthritis (Sainz et al., 2015), *A*. *platys* has not previously been associated with canine arthritis. Therefore the neutrophilic joint inflammation in this case cannot be attributed to *A*. *platys* infection, especially in view of the lack of joint fluid culture or PCR to rule out common pathogens or synovial fluid PCR to amplify *A*. *platys* DNA. Further studies may be warranted to address any potential association of *A*. *platys* and arthritis.

The phylogenetic trees of the sequences derived in this study, together with existing sequences available in Genbank, showed grouping of species derived from a range of countries in different continents, with no differentiation according to geographical origin. For example, the sequences derived from the Cypriot samples did not cluster more closely with those obtained from other countries in Europe than those reported from countries of other continents. In our study short sequences from a single gene were analysed only, and although no differences were revealed, further investigation with additional phylogeny and amplification of multiple and longer genes is needed, in order to prove that these pathogens are not specifically adapted to any particular region.

## 6 Conclusions

The current study provides the first molecular documentation for the presence of tick borne pathogens *E. canis*, *A. platys, H. canis*, *B. vogeli* and *M. haemocanis* in dogs in Cyprus. One dog was co-infected with *E. canis*, *H. canis*, *B. vogeli* and *M. haemocanis* and to the best of our knowledge this is the first report of this multiple co-infection in dogs. It further illustrates the widespread distribution of CTBP in Mediterranean countries where *R. sanguineus* sensu lato resides. Veterinarians should consider these pathogens for dogs living in or having travelled to Cyprus. Furthermore, future studies should be carried out to determine the epidemiology of CTBP in Cyprus.

## Competing interest

The authors declare that they have no competing interests.

## Funding

This was work was supported by CAEH BBSRC doctoral training grant (BB/F016662/1) and Zoetis Animal Health.

## Authors’ contributions

CA, KP and ST conceived the study and all participated in its design and coordinate the experiments. All the haematology and cytology smears were reviewed by CA and MEM. MEM and AP participated in the initial design of the study. CA, VC, KN, VIS, EIV, FS and DC extracted the DNA and performed PCR analysis. ENB and CAEH performed phylogenetic analysis on the data. CA, CAEH, ENB and ST wrote the manuscript with input from all the authors. All authors have approved the final manuscript.

## Figures and Tables

**Table 1:** PCR gene targets, primers used and results for Cases 1–3.

Target genera or species (gene)	PCR primer sequences	PCR/Sequencing (length of available sequence data) result and accession numbers		
		Case 1	Case 2	Case 3
*Babesia/Theileria* spp. (18S rRNA gene) (Matjila et al., 2008; Oosthuizen et al., 2008)	Nbab-F: 5′-AAGCCATGCATGTCTAAGTATAAGCTTTT-3′ TB-R: 5′-GAATAATTCACCGGATCACTCG-3′	*B. vogeli* (1432 bp) KU255117	Negative	Negative
*Bartonella henselae* (*alr-gcvP* intergenic spacer) (Robinson et al., 2010)	BTHalr1-F: 5′-GAGGGAAATGACTCTCTCAGTAAAAa-3′ BTHalr1-R: 5′-CAGCCAAATATACGGGCTATCCATCAA-3′	Negative	Negative	Negative
*Borrelia burgdorferi* (*ospA* gene) (Demaerschalck et al., 1995)	SL-F: 5′-AATAGGTCTAATAATAGCCTTAATAGC −3′ SL-R: 5′-CTAGTGTTTTGCCATCTTCTTTGAAAA-3′	Negative	Negative	Negative
*‘Candidatus* Mycoplasma haematoparvum’ (16S rRNA gene) (Barker et al., 2010)	CMhp-F: 5′-GGAGAATAGCAATCCGAAAGG-3′ CMhp-R: 5′-GCATTTACCCCACCAACAAC-3′	Negative	Negative	Negative
*Mycoplasma haemocanis* (16S rRNA gene) (Barker et al., 2010)	Mhc-F: 5′-GTGCTACAATGGCGAACACA-3′ Mhc-R: 5′-TCCTATCCGAACTGAGACGAA-3′	Positive	Negative	Negative
*Ehrlichia canis* (16S rRNA gene) (Dawson et al., 1996)	Primary amplification ECC-F: 5′-AGAACGAACGCTGGCGGCAAGCC-3′ ECB-R: 5′-CGTATTACCGCGGCTGCTGGCA-3′	*E. canis* (372 bp) KU255118	*E. canis* (310 bp) KX756971	Negative
	Secondary amplification “canis”: 5′-CAATTATTTATAGCCTCTGGCTATAGGA-3′ HE3: 5′-TATAGGTACCGTCATTATCTTCCCTAT-3′			
*Ehrlichia/Anaplasma* spp. (16S rRNA gene) (Parola et al., 2000)	EHR16SD-F: 5′-GGTACCYACAGAAGAAGTCC-3′ EHR16SR-R: 5′-TAGCACTCATCGTTTACAGC-3′	Positive	Positive	*A. platys* (222 bp) KU255120
*Hepatozoon* spp. (18S rRNA gene) (Inokuma et al., 2002)	Hep-F: 5′-ATACATGAGCAAAATCTCAAC-3′ Hep-R: 5′-CTTATTATTCCATGCTGCAG-3′	*H. canis* (515 bp) KF724626	*H. canis* (624 bp) KU255116	Negative
*Leishmania* spp. (kDNA) (Christodoulou et al., 2012)	LEIT2-F: 5′-CGGCTTCGCACCATGCGGTG-3′ LEIB4-R: 5′-ACATCCCTGCCCACATACGC-3′	Negative	Positive	Negative

## References

[bib0005] Altschul S.F., Gish W., Miller W., Myers E.W., Lipman D.J. (1990). Basic local alignment search tool. J. Mol. Biol..

[bib0010] Baneth G. (2011). Perspectives on canine and feline hepatozoonosis. Vet. Parasitol..

[bib0015] Barker E.N., Tasker S., Day M.J., Warman S.M., Woolley K., Birtles R., Georges K.C., Ezeokoli C.D., Newaj-Fyzul A., Campbell M.D., Sparagano O.A.E., Cleaveland S., Helps C.R. (2010). Development and use of real-time PCR to detect and quantify *Mycoplasma haemocanis* and *Candidatus* Mycoplasma haematoparvum in dogs. Vet. Microbiol..

[bib0020] Chalker V.J. (2005). Canine mycoplasmas. Res. Vet. Sci..

[bib0025] Chochlakis D., Ioannou I., Sharif L., Kokkini S., Hristophi N., Dimitriou T., Tselentis Y., Psaroulaki A. (2009). Prevalence of *Anaplasma* sp. in goats and sheep in Cyprus. Vector Borne Zoonotic Dis..

[bib0030] Chochlakis D., Ioannou I., Sandalakis V., Dimitriou T., Kassinis N., Papadopoulos B., Tselentis Y., Psaroulaki A. (2012). Spotted fever group *Rickettsiae* in ticks in Cyprus. Microb. Ecol..

[bib0035] Christodoulou V., Antoniou M., Ntais P., Messaritakis I., Ivovic V. (2012). Re-emergence of visceral and cutaneous leishmaniasis in the greek Island of crete. Vector Borne Zoonotic Dis..

[bib0040] Dantas-Torres F. (2010). Biology and ecology of the brown dog tick, *Rhipicephalus sanguineus*. Parasit. Vectors.

[bib0045] Dawson J.E., Biggie K.L., Warner C.K., Cookson K., Jenkins S., Levine J.F., Olson J.G. (1996). Polymerase chain reaction evidence of *Ehrlichia chaffeensis*, an etiologic agent of human ehrlichiosis, in dogs from southeast Virginia. Am. J. Vet. Res..

[bib0050] Demaerschalck I., Benmessaoud A., Dekesel M., Hoyois B., Lobet Y., Hoet P., Bigaignon G., Bollen A., Godfroid E. (1995). Simultaneous presence of different *Borrelia burgdorferi* genospecies in biological fluids of Lyme-disease patients. J. Clin. Microbiol..

[bib0055] Inokuma H., Okuda M., Ohno K., Shimoda K., Onishi T. (2002). Analysis of the 18S rRNA gene sequence of a *Hepatozoon* detected in two Japanese dogs. Vet. Parasitol..

[bib0060] Ioannou I., Chochlakis D., Kasinis N., Anayiotos P., Lyssandrou A., Papadopoulos B., Tselentis Y., Psaroulaki A. (2009). Carriage of *Rickettsia* spp., *Coxiella burnetii* and *Anaplasma* spp. by endemic and migratory wild birds and their ectoparasites in Cyprus. Clin. Microbiol. Infect..

[bib0065] Ioannou I., Sandalakis V., Kassinis N., Chochlakis D., Papadopoulos B., Loukaides F., Tselentis Y., Psaroulaki A. (2011). Tick-borne bacteria in mouflons and their ectoparasites in Cyprus. J. Wildl. Dis..

[bib0070] Le Riche P.D., Altan Y., Campbell J.B., Efstathiou G.C. (1974). Ticks (*Ixodoidea*) of domestic animals in Cyprus. Bull. Entomol. Res..

[bib0075] Di Loria A., Lombardi P., Avallone L., Ciaramella P. (2009). Effects of therapy on haemostasis in dogs infected with *Leishmania infantum*, *Ehrlichia canis*, or both combined. Vet. Rec..

[bib0080] Matjila P.T., Leisewitz A.L., Oosthuizen M.C., Jongejan F., Penzhorn B.L. (2008). Detection of a *Theileria* species in dogs in South Africa. Vet. Parasitol..

[bib0085] Mazeris A., Soteriadou K., Dedet J.P., Haralambous C., Tsatsaris A., Moschandreas J., Messaritakis I., Christodoulou V., Papadopoulos B., Ivović V., Pratlong F., Loucaides F., Antoniou M. (2010). Leishmaniases and the Cyprus paradox. Am. J. Trop. Med. Hyg..

[bib0090] Mekuzas Y., Gradoni L., Oliva G., Foglia Manzillo V., Baneth G. (2009). *Ehrlichia canis* and *Leishmania infantum* co-infection: a 3-year longitudinal study in naturally exposed dogs. Clin. Microbiol. Infect..

[bib0095] Mylonakis M.E., Leontides L., Gonen L., Billinis C., Koutinas A.F., Baneth G. (2005). Anti-*Hepatozoon canis* serum antibodies and gamonts in naturally-occurring canine monocytic ehrlichiosis. Vet. Parasitol..

[bib0100] Mylonakis M.E., Saridomichelakis M.N., Lazaridis V., Leontides L.S., Kostoulas P., Koutinas A.F. (2008). A retrospective study of 61 cases of spontaneous canine epistaxis (1998–2001). J. Small Anim. Pract..

[bib0105] Novacco M., Meli M.L., Gentilini F., Marsilio F., Ceci C., Pennisi M.G., Lombardo G., Lloret A., Santos L., Carrapiço T., Willi B., Wolf G., Lutz H., Hofmann-Lehmann R. (2010). Prevalence and geographical distribution of canine hemotropic mycoplasma infections in Mediterranean countries and analysis of risk factors for infection. Vet. Microbiol..

[bib0110] Oosthuizen M.C., Zweygarth E., Collins N.E., Troskie M., Penzhorn B.L. (2008). Identification of a novel *Babesia* sp. from a sable antelope (*Hippotragus niger* Harris 1838). J. Clin. Microbiol..

[bib0115] Parola Phulippe, Roux Veronique, Camicas Jean-Louis, Baradji Issa, Brouqui Philippe, Raoult D. (2000). Detection of *ehrlichiae* in African ticks by polymerase chain reaction. Trans. R. Soc. Trop. Med. Hyg..

[bib0120] Psaroulaki A., Chochlakis D., Sandalakis V., Vranakis I., Ioannou I., Tselentis Y. (2009). Phylogentic analysis of *Anaplasma ovis* strains isolated from sheep and goats using *groEL* and *mps4* genes. Vet. Microbiol..

[bib0125] Psaroulaki A., Chochlakis D., Angelakis E., Ioannou I., Tselentis Y. (2014). *Coxiella burnetii* in wildlife and ticks in an endemic area. Trans. R. Soc. Trop. Med. Hyg..

[bib0130] Robinson M.T., Morgan E.R., Woods D., Shaw S.E. (2010). Real-time and multiplex real-time polymerase chain reactions for the detection of *Bartonella henselae* within cat flea, *Ctenocephalides felis*, samples. Med. Vet. Entomol..

[bib0135] Sainz Á., Roura X., Miró G., Estrada-Peña A., Kohn B., Harrus S., Solano-Gallego L. (2015). Guideline for veterinary practitioners on canine ehrlichiosis and anaplasmosis in Europe. Parasit. Vectors.

[bib0140] Solano-Gallego L., Baneth G. (2011). Babesiosis in dogs and cats-Expanding parasitological and clinical spectra. Vet. Parasitol..

[bib0145] Tamura K., Stecher G., Peterson D., Filipski A., Kumar S. (2013). MEGA6: molecular evolutionary genetics analysis version 6.0. Mol. Biol. Evol..

